# Spontaneous regression of occult breast cancer with axillary lymph node metastasis: A case report

**DOI:** 10.1016/j.ijscr.2019.09.017

**Published:** 2019-09-20

**Authors:** Shin Takayama, Kaishi Satomi, Masayuki Yoshida, Chikashi Watase, Takeshi Murata, Sho Shiino, Kenjiro Jimbo, Akihiko Suto

**Affiliations:** National Cancer Center Hospital, Breast Surgery Division, 5-1-1, Tsukiji, Chuo-ku, Tokyo 104-0045, Japan

**Keywords:** ALND, axillary lymph node dissection, GATA3, GATA binding protein 3, ER, estrogen receptor, PgR, progesterone receptor, HER2, human epidermal growth factor receptor 2, CD8, cluster of differentiation 8, PD-L1, programmed death-ligand 1, Spontaneous regression, Breast cancer, Axillary lymphatic metastasis

## Abstract

**Introduction:**

Spontaneous regression of a malignant tumor is defined as “the partial or complete disappearance of a malignant tumor in the absence of any treatment.” Herein, we report a case of occult breast cancer with axillary lymph node metastasis that showed spontaneous tumor regression based on the histopathological findings.

**Presentation of the case:**

A 67-year-old woman presented with left armpit pain and a lump. Previous examination by another doctor revealed swelling of the left axillary lymph node, but it was difficult to identify the primary lesion. Needle biopsy of the left axillary lymph node revealed malignant tumor tissue with extensive necrosis on histological examination. On initial examination at our hospital, the left axillary lymph node was observed to have shrunk compared to previous observations. Moreover, findings indicated a suspected concentrated cyst in the left breast, with slight contrast enhancement on magnetic resonance imaging. Considering a diagnosis of occult breast cancer with axillary lymph node metastasis, excisional biopsy was performed for the left breast mass and axillary lymph node dissection for left axillary lymph node metastasis. Histological examination revealed a micro adenocarcinoma with lymphocyte infiltration in the left breast, and the viable tumor in the left axillary lymph node had disappeared. The histopathological findings of the primary tumor and dissected lymph nodes suggested the possibility of spontaneous regression of both the primary and metastatic lesions, because effective preoperative therapy was not performed.

## Highlights

• This case showed spontaneous regression of both the primary and metastasis lesions.
• Histopathological findings suggested spontaneous regression of the tumor.
• The presence of CD8-positive lymphocytes suggests involvement of the immune response.
• Poor prognostic factors of malignant tumors might induce spontaneous regression.

## 1 Introduction

Spontaneous regression of a malignant tumor is defined as “the partial or complete disappearance of a malignant tumor in the absence of treatment, or in the presence of therapy that is considered inadequate to exert a significant influence on neoplastic disease” [1]. In Japan, although the incidence of breast cancer is the highest among women and 1 of every 11 people have breast cancer, reports of spontaneous regression of breast cancer are extremely rare [2]. Hence, herein, we report a case of occult breast cancer with axillary lymph node metastasis that showed spontaneous regression of the malignant tumor considering the histopathological findings, along with a literature review. This study has been reported in line with the SCARE criteria [3].

## 2 Presentation of the case

A 67-year-old woman consulted a municipal hospital with pain and a lump in the left axilla. Needle biopsy revealed a malignant tumor on histological examination. On immunostaining, lymph node metastasis from breast cancer or accessory breast cancer was suspected. The patient was referred to another university hospital, where she was examined using various techniques including mammography, breast ultrasonography, contrast-enhanced computed tomography scan, and bone scintigraphy.

### 2.1 Ultrasonography findings at the previous hospital

**Fig. 1:**
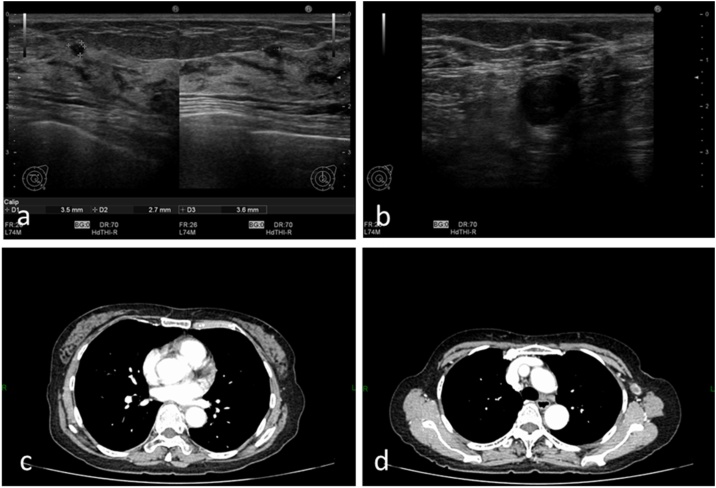
Imaging findings obtained at another university hospital. (a) Ultrasonography revealed a circular tumor in the upper outer region of the left breast, with a clear boundary and internal hypoechoic lesion. (b) Ultrasonography also revealed a circular lymph node with a 15- × 15-mm hypoechoic lesion in the left axilla. (c) Contrast-enhanced computed tomography (CT) revealed point-like signals in the upper outer region of the left breast. (d) Contrast-enhanced CT also revealed swollen lymph nodes with contrast-enhanced findings in the left axilla. The center of the lymph node showed poor contrast-enhanced findings.

In the upper outer region of the left breast, a circular tumor was observed with a clear boundary and a 3.5-mm internal hypoechoic lesion (Fig. 1a). A swollen lymph node with a 15- × 15-mm hypoechoic lesion was found in the left axilla (Fig. 1b).

### 2.2 Contrast-enhanced computed tomography findings

Contrast-enhanced point-like signals were observed in the upper outer region of the left breast (Fig. 1c). Swollen lymph nodes with contrast-enhanced findings were noted in the left axilla (Fig. 1d). The center of the lymph nodes showed poor contrast findings.

**Fig. 2:**
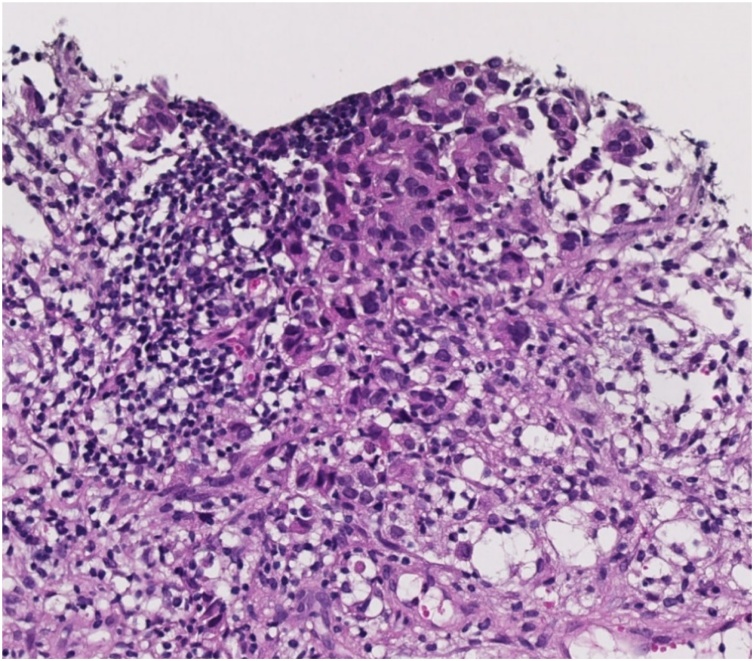
Histopathological findings of lymph node biopsy. Malignant tumor tissues showed extensive necrosis, with tumor cells that can be observed morphologically found in only a small portion.

Cancer metastasis to the left axillary lymph node was suspected, but identification of the primary tumor was difficult. Ultimately, the patient was diagnosed with unknown primary cancer and was recommended to undergo axillary lymph node dissection (ALND) and whole breast irradiation. However, she visited our hospital for a second opinion. Our pathologist reviewed the histopathological assessment of the core needle specimen. Malignant tumor tissue with extensive necrosis was observed, and tumor cells that could be observed morphologically were found in only a small portion. Tumor cells were observed with mild nuclear polymorphism and low frequency mitotic counts (Fig. 2). We explained that the pathological findings and imaging findings observed in the previous hospital result in a more obvious suspicion of unknown primary cancer rather than accessory breast cancer. We explained that ALND, chemotherapy, and radiotherapy are suitable treatments. Nevertheless, as the patient decided to undergo treatment at our hospital, we re-performed breast ultrasonography and positron emission tomography/magnetic resonance imaging (PET-MRI) at our institution.

### 2.3 Ultrasonography findings at our hospital

**Fig. 3:**
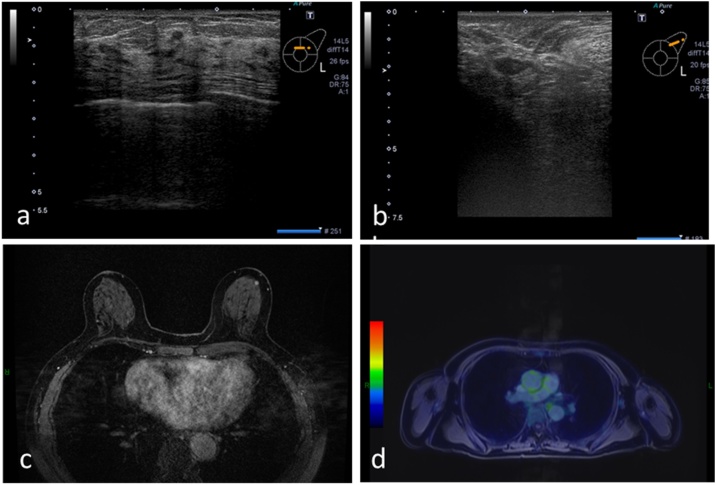
Imaging findings obtained at our institution. (a) Breast ultrasonography revealed a 3- × 2-mm mass with a clear boundary in the upper outer region of the left breast. (b) Breast ultrasonography revealed that the double structure of the left axillary lymph node disappeared and it shrunk compared to the previous findings; the lymph node now measured 15 × 7 mm. (c) Positron emission tomography/magnetic resonance imaging (PET-MRI) revealed a point-like enhancement effect with gadolinium (Gd), indicating fluorodeoxyglucose (FDG) accumulation. (d) PET-MRI revealed that FDG accumulation in the left axillary lymph node was conspicuous compared to the contralateral side.

In the upper outer region of the left breast, a 3- × 2-mm iso-echoic mass with clear boundary was observed (Fig. 3a). Doppler echocardiography confirmed blood flow at the margins, while elastography did not show a decrease in distortion. Normal lymph node structure of the left axillary lymph node disappeared, and the node shrunk compared to the previous findings (Fig. 3b); the lymph node now measured 15 × 7 mm.

### 2.4 PET-MRI findings

Point-like high signal areas were observed in the diffusion-weighted image, with a decrease in the apparent diffusion coefficient in the upper outer region of the left breast. A point-like enhancement effect was observed with gadolinium (Fig. 3c), indicating fluorodeoxyglucose accumulation. Fluorodeoxyglucose accumulation in the left axilla was quite conspicuous (Fig. 3d) compared to the contralateral side, indicating micro lymph node metastasis.

**Fig. 4:**
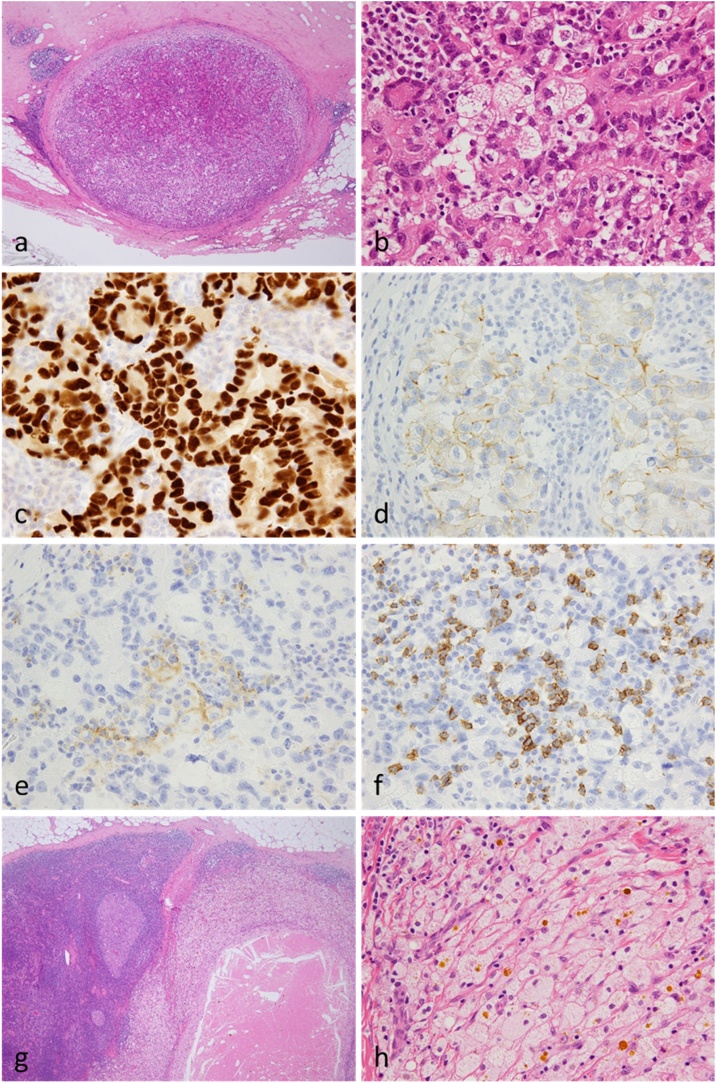
Histopathological findings of the primary tumor and dissected lymph nodes. (a) A well-defined tumor with a high degree of inflammatory cell infiltration can be observed in the breast parenchyma (hematoxylin and eosin [HE] staining, low-power magnification). (b) Tumor cells form irregular glands and are accompanied by a high degree of lymphocyte infiltration. Foamy histiocytes can be observed (HE staining, high-power magnification). Immunohistochemical staining for ER (c), HER2 (d), PD-L1 (e), and CD8 (f). (g) In dissected axillary lymph nodes, foamy histiocytes replaced part of the lymph node tissue (HE staining, low-power magnification). (h) Foamy histiocytes can be observed but no viable tumor cells are noted (HE staining, high-power magnification).

In our hospital, it is a policy to perform ALND for left axillary lymph node metastasis with a diagnosis of primary unknown cancer and excisional biopsy for diagnostic purposes of the left breast tumor. Therefore, we performed left breast tumor excision (partial breast excision) and left ALND. Finally, a 3- × 2-mm adenocarcinoma was diagnosed in the upper outer region. Histological examination revealed a well-defined tumor with a high degree of inflammatory cell infiltration in the breast parenchyma (Fig. 4a). The tumor cells formed irregular glands and showed a high degree of lymphocyte infiltration. Foamy histiocytes were often observed. The cells showed nuclear polymorphism and high mitotic counts (Fig. 4b). The histological grade was high. On immunostaining, the tumors were positive for anti-pan cytokeratin antibody (AE1/AE3), weakly positive for anti-GATA3 antibody, and strongly positive for ER (>95%; Fig. 4c), PgR (60%), and HER2 (score 2+; Fig. 4d). The breast cancer tumor was positive for PD-L1 (Fig. 4e) and the lymphocytes were positive for CD8 (Fig. 4f). Necrosis and a collection of histiocytes were observed in one of the left axillary lymph nodes (Fig. 4g). Immunohistochemistry revealed that the AE1/AE3-positive tumor cells did not metastasize. The area presumed to be the original tumor location showed foamy histiocytes instead of tumor cells (Fig. 4h). Viable malignant tumor cells disappeared from the left axillary lymph node, suggesting the possibility of spontaneous regression of the malignant tumor.

Postoperative adjuvant therapy included hormonal therapy and whole breast irradiation, and the patient is alive without recurrence 2 years after surgery.

## 3 Discussion

The definition of spontaneous regression of cancer was proposed by Everson and Cole in 1956. In that study, of 47 patients, spontaneous regression was observed in only 4 patients with breast cancer (8.5%) [1]. The incidence of spontaneous regression is extremely rare (1 in 60,000 to 100,000 cases), and it has been reported mainly in kidney cancer, neuroblastoma, malignant melanoma, and choriocarcinoma. In a review of spontaneous regression among 741 cases of cancer, only 43 cases (5.8%) of breast cancer showed regression, although the low frequency of spontaneous regression was similar to that of other common cancers such as colon cancer and lung cancer [2]. On reviewing the literature, we found a few cases of spontaneous regression of breast cancer. Possible mechanisms of spontaneous regression include the activation of CD8-positive T cells by tumor cells [4], activation of immune reactions due to trauma [5], and lymphocyte redistribution to the tumor owing to steroids [6]. The causes of spontaneous regression include involvement of the immune system and hormones, involvement of growth factors/cytokines, induction of differentiation, elimination of carcinogens, tumor necrosis, and inhibition of angiogenesis [7,8]. Among 176 malignant tumors, spontaneous regression was observed in 71 cases (41%) after surgical invasion [9]. Thus, surgical invasion causes an increased host immunity reaction that, in turn, may actually increase an individual’s natural defense against tumors. Even in the current case, needle biopsy invasion may have resulted in spontaneous regression, although the extensive necrotic tissue at the time of needle biopsy eliminates this possibility.

Carcinomas such as renal cell carcinoma, neuroblastoma, and malignant melanoma have high immune activities, suggesting that the immunological mechanism is an important factor that may result in spontaneous regression [10]. Immunogenicity is maintained in tumor cells themselves, but suppression of antitumor immunity via immunoregulatory cells is involved in cancer progression [11]. The mechanism by which cell-mediated immunity acts against tumors is being elucidated. Tumor cells express class I human leukocyte antigen on the cell membrane surface, activate cytotoxic T cells, and recognize tumor cells, thereby resulting in antitumor effects [12]. In the current case, no effective treatment was administered before the surgery; moreover, the tumor was replaced by foamy tissue and the tumor area showed invasion by CD8-positive lymphocytes, indicating that immunological reactions may be involved in spontaneous regression.

Tumor-infiltrating lymphocytes (TILs) include various types of lymphocytes. TIL activity is regulated by complex immune system activators and inhibitor pathways [13]. TILs are associated with clinical therapeutic responses in various solid tumors [14]. TIL expression in the tumor microenvironment of breast cancer is a favorable prognostic factor, particularly in triple-negative breast cancer or human epidermal growth factor receptor 2 (HER2)-positive cancer [15]. Accordingly, it is important to understand the roles of TIL, PD-1, and PD-L1 in breast cancer, as immunotherapies based on PD-1/PDL1 inhibitors are being developed. The expression of PD-L1 and PD-1 in early breast cancer is associated with higher TIL scores and pCR; conversely, the expression of these proteins is associated with poor prognostic clinicopathological factors such as tumor grade and subtype [16]. Although the tumor in the current case was of the luminal type, the histological grade and Ki-67 were high, and PD-L1 was expressed, indicating that poor prognostic clinicopathological factors might induce immunologic responses, resulting in spontaneous regression.

Here, we reported about a patient with occult breast cancer and axillary lymph node metastasis who had spontaneous tumor regression based on histopathological findings. It is important to understand the mechanism of spontaneous regression of malignant tumors, similar to this case, for further development of cancer prevention methods with cancer vaccines as well as cancer treatment with immune checkpoint inhibitors.

## Sources of funding

This research did not receive any specific grant from funding agencies in the public, commercial, or not-for-profit sectors.

## Ethical approval

The publication of this case report was exempt from approval from the ethical board of our institution.

## Consent

Written signed consent was obtained from the patient.

## Author’s contribution

Takayama designed the study and wrote the initial draft of the manuscript. Satomi and Yoshida contributed to pathological analysis and data interpretation and assisted in manuscript preparation. All the other authors contributed to data collection and interpretation and critically reviewed the manuscript. All authors approved the final version of the manuscript and agree to be accountable for all aspects of the work in ensuring that questions related to the accuracy or integrity of any part of the work are appropriately investigated and resolved.

## Registration of research studies

NA.

## Guarantor

Shin Takayama is the guarantor of this study.

## Provenance and peer review

Not commissioned, externally peer-reviewed.

## Declaration of Competing Interest

The authors declare that they have no conflict of interest.
